# Identification of the two KIT isoforms and their expression status in canine hemangiosarcomas

**DOI:** 10.1186/s12917-016-0772-y

**Published:** 2016-07-16

**Authors:** Yi-Chen Chen, Jiunn-Wang Liao, Wei-Li Hsu, Shih-Chieh Chang

**Affiliations:** Department of Veterinary Medicine, College of Veterinary Medicine, National Chung Hsing University, 250 Kuo-Kuang Road, Taichung, 40227 Taiwan; Graduate Institute of Veterinary Pathobiology, College of Veterinary Medicine, National Chung Hsing University, 250 Kuo-Kuang Road, Taichung, 40227 Taiwan; Graduate Institute of Microbiology and Public Health, College of Veterinary Medicine, National Chung Hsing University, 250 Kuo-Kuang Road, Taichung, 40227 Taiwan; Veterinary Medical Teaching Hospital, College of Veterinary Medicine, National Chung Hsing University, 250 Kuo-Kuang Road, Taichung, 40227 Taiwan

**Keywords:** Canine, Hemangiosarcoma, *c-kit*, GNSK-deletion, Isoform, KIT

## Abstract

**Background:**

KIT is a tyrosine kinase growth factor receptor. High expression of KIT has been found in several tumors including canine hemangiosarcoma (HSA). This study investigated the correlation of KIT expression and *c-kit* sequence mutations in canine HSAs and benign hemangiomas (HAs).

**Results:**

Immunohistochemistry (IHC) staining confirmed KIT expression in 94.4 % (34/36) of HSAs that was significantly higher than 0 % in HAs (0/16). Sequencing the entire *c-kit* coding region of HSAs and normal canine cerebellums (NCCs) revealed GNSK-deletion in exon 9. As for exon 9 genotyping by TA-cloning strategy, GNSK-deletion *c-kit* accounted for 48.6 % (68/140) colonies amplified from12 KIT-positive HSAs, a significantly higher frequency than 14.1 % (9/64) of colonies amplified from six NCCs.

**Conclusions:**

Due to the distinct expression pattern revealed by IHC, KIT might be used to distinguish benign or malignant vascular endothelial tumors. Moreover, the high incidence of GNSK-deletion *c-kit* in canine HSAs implicates KIT isoforms as possibly participating in the tumorigenesis of canine HSAs.

**Electronic supplementary material:**

The online version of this article (doi:10.1186/s12917-016-0772-y) contains supplementary material, which is available to authorized users.

## Background

Hemangiosarcomas (HSAs) are highly malignant tumors of vascular endothelial origin, which occur more frequently in dogs than other domestic species and are characterized by a high fatality rate [1, 2]. Surgical excision remains the primary treatment for most dogs with HSAs [3]. However, owing to a poor outcome by surgery alone, adjuvant chemotherapy is suggested for canine HSAs [4].

KIT protein, encoded by proto-oncogene *c-kit*, is a tyrosine kinase growth factor receptor for stem cell factor (SCF). Generally, KIT is activated by auto-phosphorylation upon the binding of its ligand SCF [5]; however it is constitutively expressed in a number of cells including mast cells, and hematopoietic stem cells [6]. In humans, KIT is often expressed in angiosarcomas, but it is not detected in most benign vascular tumors, and KIT positivity is more likely related to an immature phenotype [7]. As with human tissues, KIT is expressed in a large proportion of canine HSAs [8]. It has been demonstrated that activating mutations of *c-kit* typically confer constitutional KIT phosphorylation leading to downstream activation independent of ligand binding [9]. Among the 21 exons, the common activating mutations are located in exons 11 and 17, coding for juxtamembrane domain and tyrosine kinase domain of KIT, respectively [10–12]. Gain-of-function mutations have been proposed to contribute to neoplastic growth of several tumor mast cell lines [13]. Sequence analysis of canine mast cell tumors (MCTs) identified several mutations in exons 8, 9, 11, and 17 of *c-kit* RNA [14]. In contrast to MCTs, no mutation was found in these two exons (11 and 17) of *c-kit* gene in KIT-positive neoplasms of vascular cell origin [7].

Imatinib (Gleevec; Novartis), the tyrosine kinase inhibitor that targets activating mutations of *c-kit*, has been used in patients with gastrointestinal stromal tumors (GIST) harboring a *c-kit* exon 11 mutation. Decreases in tumor mass and increases of survival time have been reported [15, 16].

At present, *c-kit* gene polymorphism was detected in various canine tumors [17], but information of mutations or the impact of the *c-kit* gene in canine HSAs was limited. Since *c-kit* gene mutations could influence the expression level of KIT, and also the sensitivity of kinase inhibitor (such as imatinib mesylate) treatment [18], a systemic investigation of the mutation status of *c-kit* would provide prognostic information for tumor pathogenesis and also for the clinical response of patients under imatinib therapy [19]. Hence, this study aimed to evaluate the contribution of KIT expression and also genetic variations in the malignancy of canine HSAs.

## Methods

### Samples

Dogs with cutaneous hemangiomas (HAs) or splenic/cutaneous HSAs presented to the Veterinary Medical Teaching Hospital from 2005 to 2012 were enrolled. Medical records included breed, age, sex, number of tumors, tumor size, anatomical location, clinical history, physical examination, complete blood count, serum biochemical profile, thoracic and abdominal radiographs, abdominal ultrasonography, fine-needle cytological examination and needle core biopsy or surgical excision for histological examination. All HSAs were staged according to the World Health Organization staging system for canine splenic HSAs [9] and cutaneous HSAs [20]. The protocol of study was approved by the Institutional Animal Care and Use Committee of National Chung Hsing University [IACUC Number: 102–70].

In total, 52 specimens (16 HAs by surgical excision, 24 HSAs by surgical excision and 12 cutaneous HSAs by needle core biopsy) were included in this study. For histologic examination, specimens were fixed in neutral-buffered 10 % formalin overnight, and then processed routinely. Sections were stained with haematoxylin and eosin. Histologic grading of HSAs was assigned by cumulative scores of differentiation, mitotic rate (the number of mitotic figures per 10 high magnification fields) and percentage of necrosis [21].

### Immunohistochemistry (IHC) analysis

Serial sections of formalin-fixed and wax-embedded tissues were deparaffinized and rehydrated. Table 1 lists primary and secondary antibodies and antigen retrieval procedures used in immunohistochemical (IHC) analysis. Antigen retrieval was modified from a previous study [22]. Slides in buffer solution were cooled at room temperature for 20 min, and incubated with peroxidase-blocking reagent (S200389, Dako) for 30 min, and then treated with primary antibodies. In each interval of the following procedures, sections were rinsed with a mixture of Tris-Buffered Saline and Tween-20. Slides were then reacted with secondary antibody followed by incubation of DAB and chromogen (dilution 1 μL in 100 μL) from a commercial ChemMate EnVision detection kit (K5007, Dako). Finally, sections were counterstained with Mayer’s hematoxylin for 2 min then rinsed with DDW, and incubated with 37 mM ammonia water for 5 s and rinsed with DDW.

**Table 1:** Primary and secondary antibodies and antigen retrieval procedures used in IHC analysis of this study

					Time for incubation		
Antibody	pAb/mAb (clone)	Host	Source	Antigen retrieval (buffer, microwave, interval)	Primary Ab	Secondary Ab	DAB+ Chromagen
von Willebrand factor	pAb	Rabbit anti human	Dako	Citrate buffer (0.01 M pH 6.0),700 W-10 min and 300W-10 min	1:200, 30 min	10 min	1 min
vimentin	mAb (V9)	Mouse anti human	Dako	TE buffer (pH 9.0), 700 W-10 min and 400W-10 min	1:150, 1 h	10 min	10 min
KIT	pAb	Rabbit anti human	Dako	TE buffer (pH 9.0),700 W-10 min and 400W-10 min	1:400, 30 min	10 min	2 min

*pAb* polyclonal antibody, *mAb* monoclonal antibody, *TE buffer* Tris-EDTA buffer solution

For immunolabelingvon Willebrand factor (vWF), a canine subcutaneous granuloma acted as a positive control, and normal vessels in tissues surrounding the tumor served as an internal positive control [8]. For immunolabeling vimentin or KIT, normal adipose tissues [23] or a normal canine cerebellum (NCC) [24] was used as a positive control respectively. Replacement of primary antibody with antibody dilution buffer served as a negative control.

To score expression, this study followed an immunoreactive score (IRS) system from a previous study [25] in which IRS = SI (staining intensity) x PP (percentage of positive cells). SI was assigned as 0, negative; 1, weak; 2, moderate; and 3, strong. PP was defined as 1, <10 % positive cells; 2, 10–50 % positive cells; and 3, >50 % positive cells. Ten high magnification fields were randomly chosen for IRS evaluation. The expression level was classified as ‘negative’, ‘weak’, or ‘strong’, corresponding to IRS values of 0–1, 2–4 and 6–9, respectively. For KIT expression, three patterns were determined as pattern I, a membrane associated pattern with little to no cytoplasmic staining; pattern II, a focal (paranuclear or Golgi-like) cytoplasmic pattern with only occasional minor membrane staining, and pattern III, a diffuse cytoplasmic pattern [26].

### Identification of *c-kit* coding sequences

Total RNA extracted with Trizol (Invitrogen) from 12HSAs with KIT-overexpression and from a NCC for amplifying wild-type open reading frame of *c-kit* gene were reverse transcribed into cDNA. A solution of 2 μg RNA, 2 μL of 5 μM random primers, 4 μL of 2.5 mM dNTP and 7 μL DDW were incubated at 65 °C for 5 min, then chilled on ice for 5 min. The mixture of reverse transcriptase solution, which included 1 μL of 0.1 M dithiothreitol, 1 μL reverse transcriptase (Superscript III, Invitrogen), 4 μL of 5× First Strand Buffer (Invitrogen) and 1 μL RNase inhibitor (Promega), was added and heated at 50 °C for 60 min, then inactivated at 70 °C for 15 min and chilled on ice.

Sequencing of the entire *c-kit* coding region was determined by PCR using several sets of primers followed by automated sequencing. Primers were designed according to a previously published canine *c-kit* cDNA sequence [27]. Sequences, as well as locations of primers, are listed in Table 2.

**Table 2:** Sequences and locations of *c-kit* primers used in this study

Designation	Sequence (5′-3′)	Primer location
1F	CGATGAGAGGCGCTCGC	27–34
1R	GGCGTAACACA TGAACACTCCAG	891–913
2F	GCTGGCATCATGGTGACTTC	819–838
2R	CATGGGTTTCTGTAGATACTTGTAGG	1668–1693
3F	CACACCTTTGCTGATTGGCT	1591–1610
3R	GATTCGACCATGAGTAAGGAGG	2419–2440
4F	GGGTATGGCATTCCTGGC	2359–2376
4R	GCTTCACACATCTTCGTGTACCA	2937–2959
AF	GCTCAGAGTCTATCGCAGCCACCG	3–26
AR	CTGCCTTCTCTGTGATCCATTCGTTG	271–296
BF	GCTGTCCAAGAAATTCACCCTG	619–640
BR	ATATTACTTTCATTGTCAGACTTGGG	1124–1149
CF	AAAACTCGTCTCTGTCACCGTCTG	1422–1445
CR	GATCTCCTCAACAACCTTCCACTG	1691–1714
DF	AAATCAGAGTTAATAGTCAGTGTCGG	133–158
DR	TTTATCCACATCGAGTCCACG	729–749
EX9F	CAACAATGTAGGCAGGAGTTCTG	1495–1517
EX9R	CAGCAAAGGTGTGAACAGGG	1572–1591

*F* forward, *R* reverse

Annealing temperature dependent on combination of the primers: 55 °C for 1F and 1R, 56 °C for 2F and 2R, 52 °C for 3F and 3R, 61 °C for AF and AR, 54 °C for BF and BR, 57 °C for CF and CR, 54 °C for DF and DR, 57 °C for AF and BR, 55 °C for P2F and CR, 56 °C for CF and P3R, and 54 °C for EX9F and EX9R

In general, PCR solution included 2.5 μL of 10× Taq buffer, 2.5 μL of 2.5 mM dNTP, 1 μL of 5 mM of both sense and anti-sense primer, 0.5 U DreamTaq DNA polymerase (EP0702, Fermentas), 2 μl of RT reaction mixture and 15.5 μL DDW. PCRwas performed by carrying outinitial denaturation for 5 min at 95 °C, followed by 40 thermocycles: 1 min at 95 °C, annealing for 45 s at 52 to 62 °C (Table 2) and then 1 min at 72 °C, and final polymerization for 7 min at 72 °C. Amplified products were electrophoresed with 1.5 % agarose gel and visualized with a UV illuminator.

The amplified fragments with expected size were purified by a commercial gel extraction kit (DF300, Qiagen), which was performed with an automated sequencing(Mission Biotech Co., Taipei, Taiwan). The sequences obtained from a NCC were initially compared with a canine *c-kit* sequence [GenBank: AF448148]. Sequences of HSAs were aligned with those of NCC using the BLAST program on the NCBI.

#### Cloning of PCR amplicon containing the exon 9 region of *c-kit*

To clarify sequences of the most 3′end of *c-kit* exon 9 of HSAs, DNA fragments containing exon 9 were amplified by PCR with primers set EX9F/R (Table 2) and were then purified. Ligation was conducted by a TOPO TA Cloning Kit (450641, Invitrogen) followed by transforming to TOP10 competent cells and plating onto an LB plate containing ampicillin as well as X-gal and IPTG for the case of blue/white screening.

After 16 h of incubation, 10 to 14 white colonies randomly picked from each plate were cultured at 37 °C overnight and then the plasmid DNA was extracted via High-Speed Plasmid Mini Kit, (PD300, Geneaid). Plasmids with insertion of *c-kit* DNA were initially confirmed by the digestion pattern using are striction enzyme *Eco*RI (New England Biolabs Inc.) followed by automated sequencing (Mission Biotech Co.).

#### Statistical analysis

The statistical analysis was performed using Statistical Package for the Social Science (Version 19.0, SPSS Taiwan Corp). Univariate logistic regression was used to establish the correlation between variables and KIT immunolabeling patterns. *P*-value <0.05 indicated a statistically significant difference between categorized groups.

## Results

### Animals

As listed in Additional file 1: Table S1, of the 16 dogs with HAs, seven were male and nine were female. Dogs with HSAs included 24 males and 12 females. Pedigree dogs comprised eight breeds and accounted for 56.3 % (9/16) of dogs with HAs, and 58.3 % (21/36) of dogs with HSAs, which included Maltese (*n* = 3), Pomeranian (*n* = 2), and Golden Retriever (*n* = 2) with HAs, and Golden Retriever (*n* = 8), Miniature Schnauzer (*n* = 5), Beagle (*n* = 2), Maltese (*n* = 2), and Labrador Retriever (*n* = 2) with HSAs. Labrador retriever and Shetland sheepdog each accounted for 1/16 with HA; and Caucasian sheepdog and Welsh Corgi each accounted for 1/36 with HSA. The other 43.7 % (7/16) of HA and 41.7 % (15/36) of HSA occurred in mixed dogs. The median age in dogs with HA was 11.8 years (range 2–16 years), and 13.4 years (range 4–18 years) in dogs with HSA.

Clinical stages of HSA included stage III in 58.3 % (21/36), stage II in 22.2 % (8/36), and stage I in 19.5 % (7/36) of dogs. Histological grades were assigned as grade I in 5.6 % (2/36), grade II in 33.3 % (12/36), and graded III in 61.1 % (22/36) of dogs.

### Detection of KIT expression by IHC

**Fig. 1:**
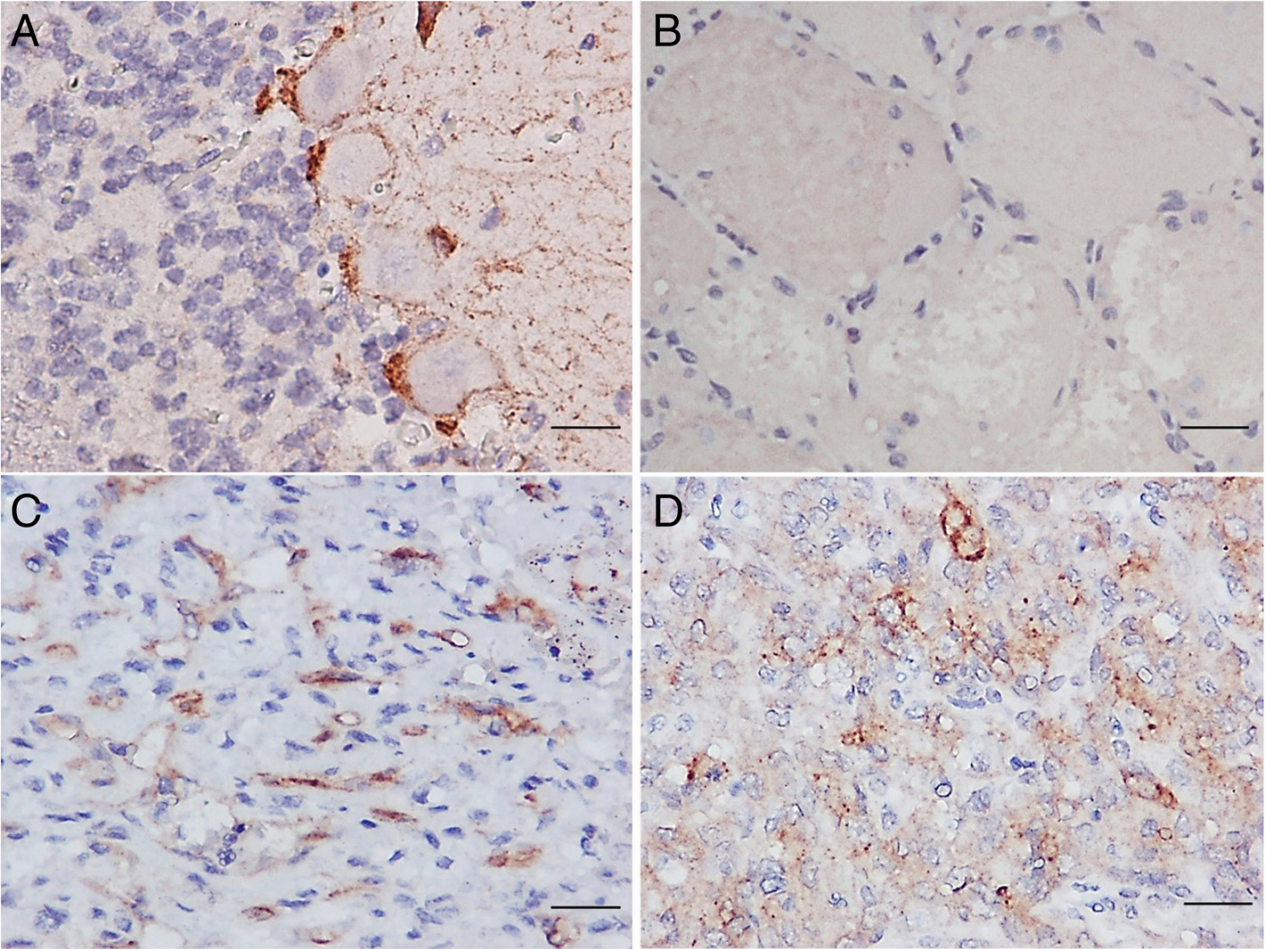
Immunohistochemical staining for KIT. **a** Strong immunolabelling in the cytoplasm of Purkinje cells of a NCC was used as a positive control for KIT immunostaining. **b** Expression of KIT was not detected in a HA. **c** Strong immunoreactivities were observed inasplenic HSA with stage III and grade 3. **d** Positive immunoreactivities were observed ina cutaneous HSA with stage III and grade 2. Sections counterstained with hematoxylin. Bar = 50 μm

To study the correlation of KIT expression with *c-kit* sequence mutations in canine HSAs, initially, KIT expression in canine HSAs and KIT expression in HAs were detected by IHC analysis, of which, KIT staining was optimized using NCC as a positive control (Fig. 1a), and omitting KIT antibody as a negative control. As listed in Table 3, vWF expression was demonstrated in all HSAs (36/36, 100 %), vimentin expression was shown in 97.2 % of HSAs (35/36), and KIT expression was detected in 94.4 % of HSAs (34/36), whereas negative KIT expression was found in all HAs (16/16, 100 %, and Fig. 1b). All KIT-positive HSAs (34/34, 100 %) showed a diffuse cytoplasmic immunostaining pattern (Fig. 1 panels c-d).

**Table 3:**
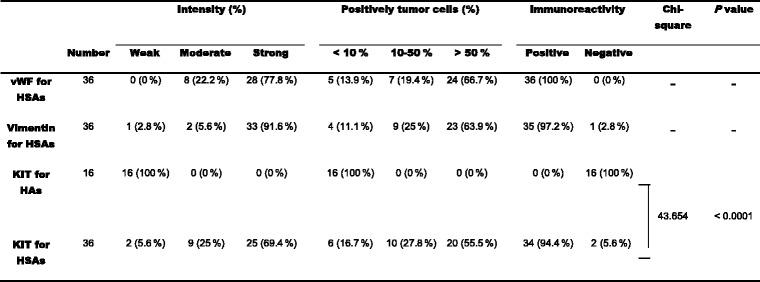
IHC analysis of vWF, vimentin and KIT expression in canine hemangiomas and hemangiosarcomas

*vWF* von Willebrand factor, *HSAs* hemangiosarcomas, *HAs* hemangiomas, *Weak* staining much weaker than positive control or negative, *Moderate* staining slightly weaker than positive control, *Strong* staining equal to positive control. -, not done. *P values* <0.05 indicate significant difference

### Identification of *c-kit* gene sequences of HSA

In order to identify possible mutations of the *c-kit* gene in canine HSAs, amplicons covering the entire coding region of the *c-kit* gene were amplified by PCR using several sets of primers followed by automated sequencing. Initially, sequences representing wild type *c-kit* gene were amplified from a NCC. Compared with the *c-kit* coding sequences [GenBank: AF448148], a G to A transversion (G^1275^A), a silent mutation at codon 425 of KIT, was identified in our NCC sample.

Next, the full length *c-kit* coding region of 12 HSAs with high KIT expression level (samples were indicated in Additional file 1: Table S1) was amplified for sequence identification. Compared with the wild-type *c-kit* sequences, the 1275 nucleotide (nt) was either A or G; 50 % (6/12) of HSAs harbored the A^1275^G substitution. In addition, 8.3 % (1/12) of HSA samples contained 3 other genetic variations at C^159^T (codon 53), C^414^T (codon 138), and A^507^G (codon 169) of *c-kit*. Overall, these four point mutations were silent mutations that did not alter amino acids.

**Fig. 2:**
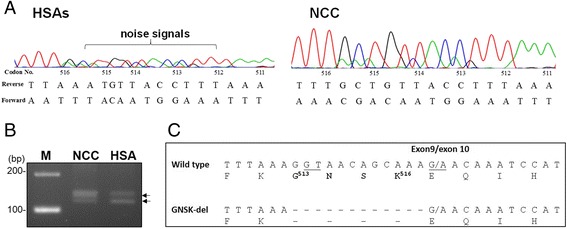
Identification of sequence variations in*c-kit*transcript of canine HSAs. Following PCR amplification, sequences of amplicons were revealed by automated sequencing. **a** Distinct sequencing chromatograms of *c-kit* exon 9 were revealed between NCCand HSA samples. **b** The PCR products containing *c-kit* exon 9 were amplified from a NCCor a HSA and resolved in 3 % agarose gel. M, 100-bp DNA size markers. **c** Sequence alignment of two *c-kit* exon 9 variants. Dashed lines indicate deletion of nucleotides. Underlines indicate the deduced conserved di-nucleotide of splicing donors and acceptor sites

Interestingly, as judged by sequencing chromatogram, overlapping noise signals starting from codon 513 to 516 (the most 3′ end) of the *c-kit* exon 9 were found in all DNA fragments amplified from HSAs (12/12, 100 %) (Fig. 2a, left panel), indicating the presence of sequence polymorphisms. This phenomenon was not found in those amplified from NCCs (Fig. 2a, right panel). Isolation of the PCR amplicons containing this region revealed two distinct products (Fig. 2b, indicated as arrowheads). Sequence alignment indicated that 12 nucleotides, coding for residues 513–516 (GNSK), were deleted from the exon 9 variant, as compared with that of wild type (Fig. 2c).

### Investigation of sequence variations in exon 9 of *c-kit*

**Fig. 3:**
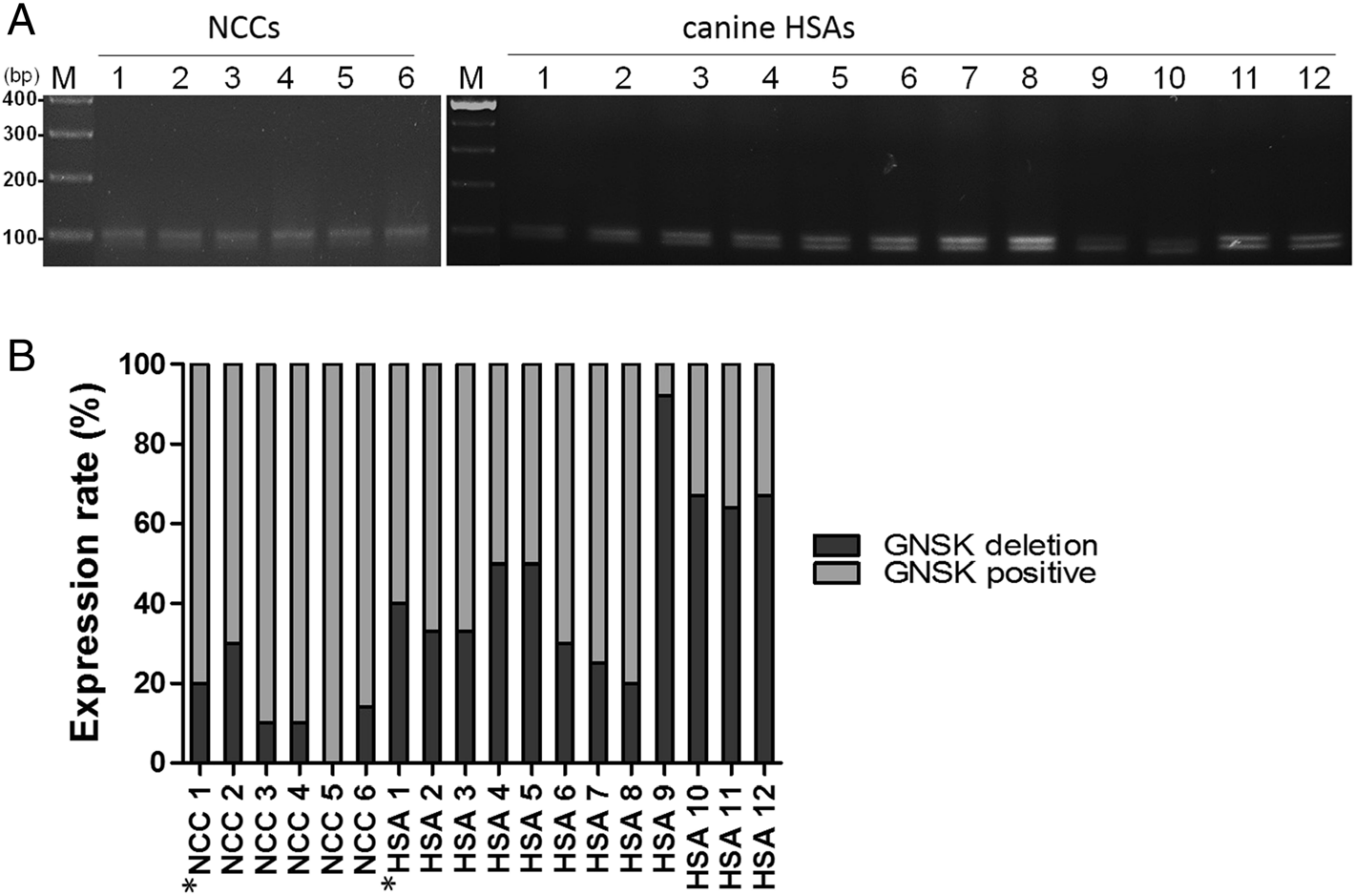
The frequency of *c-kit* exon 9 variants in canine tissues. **a** PCR products containing the exon 9 region amplified from 6 healthy canine cerebellums (*left* panel) or 12 canine HSAs (*right* panel) were electrophoresed with 3 % agarose gel. **b** The approximate frequency of the *c-kit* exon 9 variants was determined by cloning strategy. Numbers of colonies containing genotype representing wild type (*grey bar*), or 12-nt del (*black bar*) of exon 9 were summarized

Based on the migration pattern of gel electrophoresis, two types of exon 9 amplicons were present in all HSA samples (12/12), and in 83.3 % (5/6) of NCCs (Fig. 3a). It is worthy of noting that the 12 nt deletion variant, designated GNSK-deletion herein, was the major product in HSAs samples that is distinct from normal canine tissues, which is consistent with sequencing chromatogram as indicated in Fig. 2a. Next, we investigated the correlation of the *c-kit* GNSK-deletion variant with canine HSAs.

To this end, we determined the approximate frequency of these two genotypes of *c-kit* exon 9 by cloning strategies [28]. DNA amplicons were cloned into TA vector following PCR, and more than ten bacterial colonies with *c-kit* exon 9 insertion randomly picked from individual cases were sequenced. In total, sequences of 140 clones containing exon 9, amplified from 12 HSA patients and 64 clones from 6 healthy canine cerebellums, were determined. The frequency of two *c-kit* exon 9 genotypes in each sample are shown in Fig. 3b. Overall, 72 colonies from HSAs and 55 colonies from NCCs resembled wild-type *c-kit* exon 9 sequences, whereas 68 clones from HSAs and 9 clones from NCCs were the GNSK-deletion. Overall, frequency of this *c-kit* RNA variant in HSAs was 48.6 % (68/140), significantly higher than 14.1 % (9/64) of NCCs (*P* = 0.004).

## Discussion

The expression profile and the mutation status of KIT have been intensively studied in human tumors, in particular, GISTs and MCTs [29]. Mutational hot spots in different regions of the *c-kit* gene have been found in human tumors. However, little is known about the *c-kit* mutation status of canine tumors. In the present study, the frequency and location of *c-kit* gene mutations of canine HSA was investigated. We analyzed the entire coding region of the *c-kit* transcript and revealed a variant with a 12-nt deletion in exon 9. Previously, the GNSK deletion was detected in C2 canine malignant mast cell line, but not in NCC [30]. In addition, while preparing this manuscript, *c-kit* sequences of canine HSA was revealed [17]. In that study, Gramer et al., referred GNSK insertion (the full length *c-kit*) was not amplified in canine HSA, indicating GNSK-deletion allele possibly is the major transcript that is consistent with our findings. We further demonstrated, in fact, both wild type (full length) and GNSK-deletion genotypes were present in canine tumor samples. Interestingly, GNSK- deletion was present in a significantly higher level in canine HSAs as compared with NCCs.

It is worthy of noting that previously identified high frequency mutations were not present in canine HSAs; for instance, mutations in exon 11 [31], and alterations in exon 9 of *c-kit* including SNPs and duplication in human GISTs [32, 33], and SNPs (missense mutations; S^479^I, N^508^I substitution) in canine MCTs [7]. Interestingly, the GNSK-deletion presents not only in HSAs, but also in NCCs (to a much lower frequency); this variation was regarded as a *c-kit* isoform. Deletion of 12-nt (GGTAACAGCAAA, coding for G^513^NSK^516^ residues) has not been reported in previous studies on canine GISTs and MCTs [31, 34]. Based on the signal intensity of sequence chromatography and also the frequency estimated from TA cloning strategy, we suspect the two genotypes (wild type and GNSK-deletion) of KIT were expressed at similar levels in canine HSAs.

Furthermore, as GNSK-del mutation of exon 9 was not detected in genomic DNA of all samples, the presence of two *c-kit* RNAs is very likely due to alternative splicing during processes of pre-mRNA. Indeed, the deleted sequences (5′-G*GT*AACAGCAA*AG*-3′) in exon 9 comprised consensus splicing signals [35], i.e. the two nucleotides in italic font, of which the GT di-nucleotide at the 5′end, and AG at the3′ end (G residue is the first nucleotide in the adjacent exon 10) serves as a donor site and acceptor site of the splicing event, respectively. Therefore, the presence of GT and AG di-nucleotides in the end of exon 9 would act as an alternative splicing signal leading to deletion of the 12 nucleotides.

In previous studies, the isoform with deletion of a tetrapeptide sequence (GNNK) in the extracellular juxtamembrane region of KIT has been demonstrated in mice and humans [36, 37]. While these two KIT isoforms share similar affinity to ligand SCF [24], expression of KIT with GNNK deletion induced stronger transformation effects in NIH3T3 cells and was more tumorigenic in nude mice, as compared with wild type KIT [38]. The underlying mechanism has been proposed; the GNNK negative isoform can be phosphorylated (on tyrosine) at a higher level and more rapidly than the GNNK-positive isoform upon SCF ligand binding that leads to stimulating a stronger downstream signaling [39]. Additionally, the GNNK negative isoform was more resistant to tyrosine kinase inhibitor when compared with the GNNK positive isoform in the absence of human SCF [40]. Moreover, it is worthy of noting that genotyping in this study revealed the presence of GNSK-deletion in NCCs, although to a lesser extent than in canine HSAs. This is in line with the results of IHC, in which malignant HSAs and NCCs expressed high levels of KIT. Expression of KIT in the cerebellum implicates the importance of KIT in maintenance of cerebellar functionality. Indeed, embryonic knockdown expression of KIT in the rat cortex indicates KIT participates in radial migration of cortical neurons and also in the correct formation of callosal projection neurons [41]. In addition, as seen in Fig. 3, the ratio of GNSK-deletion of *c-kit* transcripts in HSAs was significantly higher than that in NCCs, indicating the role that the GNSK-deletion isoform plays in the development of HSAs, possibly due to activating downstream signaling of KIT.

The results of IHC demonstrated that KIT is predominantly expressed in malignant canine HSAs while it is absent in benign HAs. This finding is consistent with one previous study [8]. In addition, expression of KIT in all canine HSAs displayed diffuse cytoplasmic staining, even in HSAs with various histologic grades. According to the definition of a previous report, the immunostaining pattern of KIT in canine HSAs was classified into pattern III [42]. It has been reported that pattern III is significantly associated with a high histologic grade, poor differentiation [43], local recurrence, and metastasis [42].

Although most HSAs expressed high levels of KIT, the downstream signal transduction pathways activated by KIT in canine HSAs remain unclear. The active and dysregulated dimeric KIT may result in neoplastic transformation of normal cells overexpressing KIT [13]. The constitutional phosphorylation of KIT and activations of the downstream signaling pathways including the MAPK pathway, PI3K/AKT/mTOR pathway and JAK/STAT pathway have been reported [41, 44, 45]. Therefore, the role of GNSK-deletion KIT in the activation of downstream signaling pathways in canine HSAs or in the pathogenesis of canine HSAs warrants further investigation.

## Conclusions

The positive immunoactivity of KIT was demonstrated in the majority of HSAs, while it was not detectable in HAs. Hence, expression of KIT might be used as a marker to distinguish benign or malignant vascular endothelial originated tumors. Sequencing of the entire coding region indicated previously reported sequence variations were not present in canine HSAs analyzed in this study. Instead, a KIT isoform with deletion of GNSK, generated by alternative splicing, was detected in all canine HSAs tested. Moreover, a significantly higher proportion of this GNSK-deletion isoform in canine HSAs than in normal cerebellum was demonstrated for the first time.

## Abbreviations

GIST, gastrointestinal stromal tumors; HA, hemangioma; HSA, hemangiosarcoma; IHC, immunohistochemistry; IRS, immunoreactive score; MCT, mast cell tumor; NCC, normal canine cerebellum; PP, percentage of positive cell; SCF, stem cell factor; SI, staining intensity; vWF, von Willebrand factor

## Additional file

Additional file 1: Table S1. Summary of clinical characteristics of dogs with HSA or HA, and IRS of KIT expression profile. (DOCX 17 kb)
