# Supplementary material for: Identification of the two KIT isoforms and their expression status in canine hemangiosarcomas
Source: BMC Vet Res. 2016 Jul 16;12:142. doi: 10.1186/s12917-016-0772-y (PMC4947345; doi:10.1186/s12917-016-0772-y)
Supplement: Additional file 1: Table S1. — Summary of clinical characteristics of dogs with HSA or HA, and IRS of KIT expression profile. (DOCX 17 kb) [file 12917_2016_772_MOESM1_ESM.docx]

**Additional file 1: Table S1. Summary of clinical characteristics of dogs with HSA or HA, and IRS of KIT expression profile**

| Case | Breed | Sex | Age (Years) | Type | IRS of KIT | |
| --- | --- | --- | --- | --- | --- | --- |
|  |  |  |  |  | SI | PP |
| 1* | Mixed | F | 13 | cHSA | 3 | 3 |
| 2* | Mixed | M | 12 | cHSA | 3 | 3 |
| 3* | Mixed | M | 16 | sHSA | 2 | 3 |
| 4* | Golden Retriever | M | 9 | cHSA | 3 | 3 |
| 5* | Labrador Retriever | M | 12 | sHSA | 3 | 3 |
| 6* | Mixed | Mc | 14 | sHSA | 3 | 3 |
| 7* | Golden Retriever | M | 15 | sHSA | 3 | 3 |
| 8* | Golden Retriever | Mc | 4 | cHSA | 3 | 3 |
| 9* | Miniature Schnauzer | M | 13 | sHSA | 3 | 3 |
| 10* | Mixed | M | 16 | cHSA | 3 | 3 |
| 11* | Mixed | Fs | 17 | sHSA | 2 | 3 |
| 12 | Miniature Schnauzer | M | 15 | sHSA | 3 | 3 |
| 13 | Mixed | F | 14 | sHSA | 3 | 3 |
| 14 | Beagle | F | 15 | cHSA | 1 | 1 |
| 15 | Mixed | M | 16 | sHSA | 2 | 3 |
| 16 | Golden Retriever | M | 9 | cHSA | 2 | 2 |
| 17 | Mixed | M | 16 | cHSA | 2 | 3 |
| 18 | Miniature Schnauzer | Mc | 14 | sHSA | 3 | 1 |
| 19 | Miniature Schnauzer | F | 11.5 | cHSA | 3 | 3 |
| 20 | Miniature Schnauzer | M | 15 | sHSA | 3 | 2 |
| 21 | Golden Retriever | Fs | 8.5 | cHSA | 2 | 3 |
| 22 | Golden Retriever | M | 9 | cHSA | 2 | 3 |
| 23 | Maltese | Fs | 16 | cHSA | 2 | 1 |
| 24 | Mixed | Fs | 12 | cHSA | 3 | 2 |
| 25 | Mixed | Fs | 14 | sHSA | 3 | 3 |
| 26 | Mixed | M | 15 | cHSA | 2 | 3 |
| 27 | Mixed | M | 13 | cHSA | 3 | 3 |
| 28 | Golden Retriever | M | 12 | sHSA | 3 | 3 |
| 29 | Caucasian sheepdog | Mc | 15 | cHSA | 0 | 0 |
| 30 | Mixed | Fs | 15.5 | cHSA | 2 | 3 |
| 31 | Mixed | F | 18 | sHSA | 3 | 3 |
| 32 | Welsh Corgi | M | 13.5 | cHSA | 3 | 3 |
| 33 | Golden Retriever | M | 10 | cHSA | 2 | 3 |
| 34 | Labrador Retriever | M | 13 | sHSA | 1 | 2 |
| 35 | Beagle | M | 16 | sHSA | 2 | 3 |
| 36 | Maltese | Fs | 14.5 | cHSA | 2 | 3 |
| 37 | Mixed | M | 12.25 | HA | 0 | 0 |
| 38 | Mixed | F | 14 | HA | 0 | 0 |
| 39 | Mixed | F | 13 | HA | 0 | 0 |
| 40 | Mixed | F | 15 | HA | 0 | 0 |
| 41 | Pomeranian | F | 13.5 | HA | 0 | 0 |
| 42 | Maltese | F | 14 | HA | 0 | 0 |
| 43 | Shetland sheepdog | Fs | 16 | HA | 0 | 0 |
| 44 | Mixed | M | 13 | HA | 0 | 0 |
| 45 | Mixed | F | 15 | HA | 0 | 0 |
| 46 | Maltese | F | 14 | HA | 0 | 0 |
| 47 | Pomeranian | M | 2 | HA | 0 | 0 |
| 48 | Maltese | Mc | 15 | HA | 0 | 0 |
| 49 | Labrador Retriever | F | 11 | HA | 0 | 0 |
| 50 | Golden Retriever | Mc | 6 | HA | 0 | 0 |
| 51 | Golden Retriever | M | 8 | HA | 0 | 0 |
| 52 | Mixed | M | 7 | HA | 0 | 0 |

M: male, Mc: male neutered, F: female, Fs: female spayed, cHSA: cutaneous hemangiosarcoma, sHSA: splenic hemangiosarcoma, HA: hemangioma, IRS: immunoreactive score, SI: staining intensity, PP: positive percentage. *indicates sequences of *c-kit* transcript of these samples were further determined.
